# Concomitant pyogenic spondylodiscitis and empyema following tongue cancer resection and wisdom tooth extraction: A case report and literature review

**DOI:** 10.1097/MD.0000000000039087

**Published:** 2024-07-26

**Authors:** Kunio Yoshizawa, Takashi Yagi, Tsuyoshi Uchida, Takeshi Moriguchi, Akinori Moroi, Koichiro Ueki

**Affiliations:** a Department of Oral and Maxillofacial Surgery, Interdisciplinary Graduate School of Medicine, University of Yamanashi, Chuo City, Yamanashi, Japan; b Departments of Neurosurgery, University of Yamanashi, Chuo City, Yamanashi, Japan; c Department of General Thoracic Surgery, University of Yamanashi, Chuo City, Yamanashi, Japan; d Department of Emergency and Critical Care Medicine, School of Medicine, University of Yamanashi, Chuo City, Yamanashi, Japan.

**Keywords:** empyema, infection, laminectomy, oral surgery, polymyalgia rheumatica, pyogenic spondylodiscitis

## Abstract

**Rationale:**

Pyogenic spondylodiscitis is an infectious spinal disease that causes significant motor dysfunctions. Its diagnosis can be challenging owing to its rapid onset and nonspecific symptoms.

**Patient concerns:**

A 79-year-old Japanese man with a history of type 2 diabetes mellitus and polymyalgia rheumatica presented to our department with tongue pain. Following partial glossectomy and wisdom tooth extraction under general anesthesia, on 10 postoperative day (POD) the patient developed right-sided abdominal pain and difficulty in walking. On 12 POD, the patient was admitted to a municipal hospital due to respiratory distress and paraplegia.

**Diagnoses:**

The patient was diagnosed with pyogenic spondylodiscitis and empyema. Blood tests revealed elevated C-reactive protein levels (36.5), white blood cell count (19,570), and neutrophil count (17,867).

**Interventions:**

The patient received meropenem hydrate 3 g/2 days as empiric antibiotic treatment for acute infection. Upon admission to the emergency department on 16 POD, the lung abscess was drained, hemilaminectomy was performed.

**Outcomes:**

Blood cultures, sputum tests, and cultures from the thoracic and spinal abscesses drained during surgery revealed methicillin-sensitive *Staphylococcus aureus*. The infection was successfully managed, and the respiratory disturbance and inflammatory response improved. However, the lower half of the patient body remained paralyzed. Subsequently, the patient was transferred to a rehabilitation facility on 45 POD. The patient continued to undergo functional restoration training, gradually regained function, and eventually achieved the ability to walk with grasping gait.

**Lessons:**

This is the first case report of *S aureus* causing pyogenic spondylodiscitis and empyema due to blood stream infection from a post-oral surgical wound. Pyogenic spondylodiscitis arising from a secondary hematogenous infection is difficult to diagnose and can lead to severe functional impairment. Prompt and appropriate diagnosis and treatment based on detailed patient interviews, additional blood tests, and computed tomography are essential.

## 1. Introduction

Pyogenic “spondylodiscitis,” a combination of spondylitis and discitis or pyogenic spondylitis, is a rare but severe infectious disease of the spine that causes motor dysfunction. Despite its rapid progression, diagnosis can be challenging owing to its nonspecific findings and rapid onset.^[1]^ *Staphylococcus aureus* (SA) is the most common causative organism, accounting for 53.5% of the total number of cases, but other bacteria can also be responsible.^[2]^ Interestingly, SA is the second leading cause of empyema after *Streptococcus pneumoniae*.^[3]^ This report details an exceptionally rare case of concomitant pyogenic spondylodiscitis and empyema arising as a secondary infection following oral surgery. Methicillin-sensitive SA (MSSA) was identified at both infection sites and in blood cultures on 12 postoperative day (POD). Although a few cases involve *Fusobacterium spp*., *Mycobacterium tuberculosis*, and *Streptococcus milleri* as causative agents of both spondylodiscitis and empyema, none have reported SA.^[4]^

Oral bacteria can enter the bloodstream and cause systemic infections through 3 primary pathways^[5]^:

1. Bacteria can enter the bloodstream directly through affected periodontal and endodontic sites.^[6,7]^
2. During general anesthesia, bacteria can be inhaled into the lower respiratory tract, leading to infection.^[8,9]^
3. Oral bacteria can alter the intestinal microflora in the gastrointestinal tract, potentially causing adverse effects.^[10,11]^

In the present case, both pericoronitis and oral surgery with endotracheal intubation under general anesthesia provided multiple pathways for oral bacteria to cause systemic infections.

## 2. Case report

**Figure 1.**
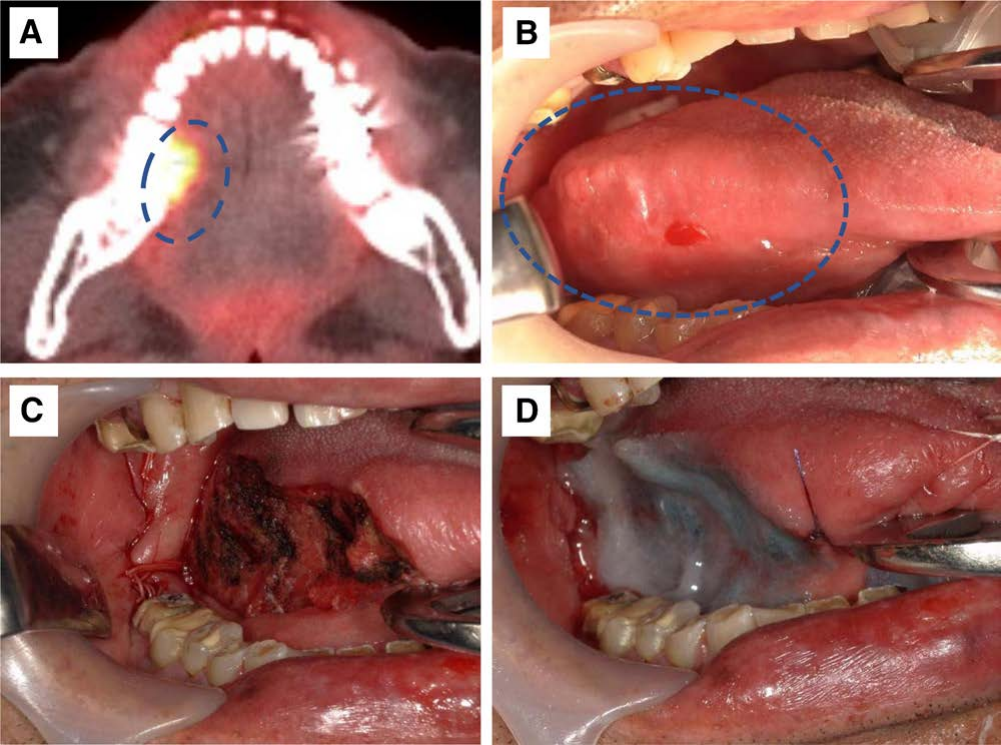
Positron emission tomography-computed tomography finding and intraoperative image. (A) The image shows an accumulation of standardized uptake value within the dotted blue circle. (B) An exophytic mass is observed within the dotted blue circle. (C) The lower right wisdom tooth extraction site and the resected tongue with a safety margin are shown. (D) The tongue wound is covered with an absorbable membrane made of polyglycolic acid material.

**Figure 2.**
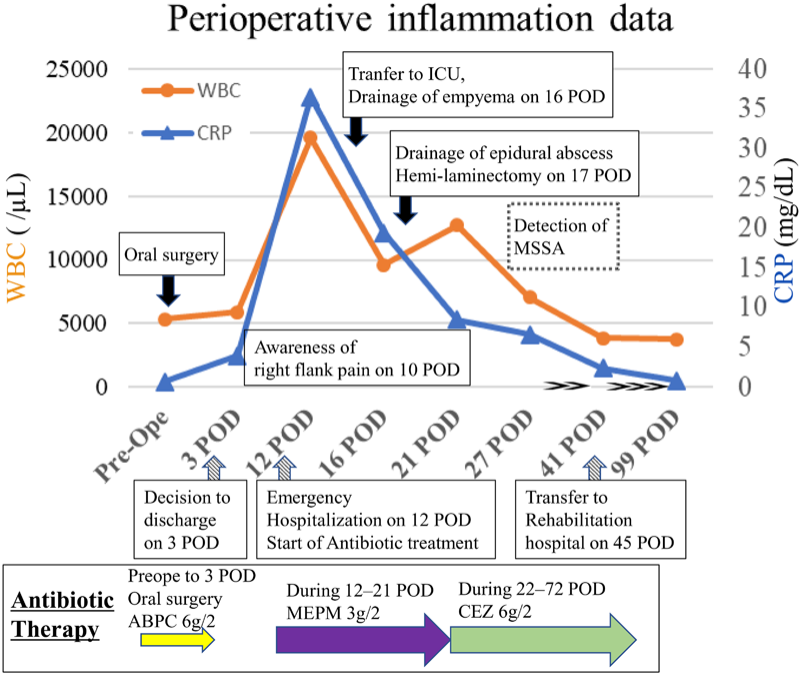
Perioperative inflammatory data and clinical course. The white blood cell count on 21 POD was temporarily elevated after laminectomy on POD 17. Double-headed arrows between PODs 27 and 41 and triple-headed arrows between PODs 41 and 99 denote compression of the time axis for brevity. POD = postoperative day after oral surgery.

A 79-year-old Japanese man presented to our department with erosion of the right side of the tongue. Panoramic radiographs revealed bone resorption around the wisdom tooth in the right mandible, which was consistent with pericoronitis. Positron emission tomography-computed tomography showed a 2-cm exophytic mass with a standardized uptake value of 10.5 at the right lingual margin (Fig. 1A and B). Due to the presence of polymyalgia rheumatica and type 2 diabetes mellitus (DM), along with a history of lacunar infarct 13 years prior, the patient was treated with prednisolone (steroid), vildagliptin/metformin hydrochloride, teneligliptin hydrobromide hydrate/canagliflozin hydrate (for DM), and clopidogrel sulfate (antiplatelet agent), effectively managing the conditions. The patient underwent partial glossectomy and wisdom tooth extraction under general anesthesia. Partial tongue resection included an area devoid of Lugol stain, and intraoperative rapid pathological examination confirmed the presence of negative margins (Fig. 1C). The surgical wound was covered with a polyglycolic acid sheet (NEOVEIL^®^, GUNZE Ltd., Osaka, Japan) and secured using a sprayed fibrin glue (Fig. 1D). To prevent infections during the perioperative period, intravenous ampicillin sodium/sulbactam sodium was administered 1 hour prior to surgery and continued until 3 POD upon discharge. The limited hospital beds due to the ongoing coronavirus disease 2019 (COVID-19) pandemic have necessitated earlier discharge. Typically, patients with postoperative tongue cancer remain hospitalized for approximately 10 days until their condition stabilizes. However, in the present case, the patient was discharged on POD 3 following the resumption of oral intake. On 10 POD, the patient experienced right-sided abdominal pain and difficulty in walking, but did not seek medical attention. Subsequently, the patient condition gradually deteriorated. On 12 POD, the patient was admitted to a municipal hospital due to respiratory distress and paraplegia. Blood tests revealed elevated C-reactive protein (CRP) levels (36.5), white blood cell count (19,570), and neutrophil count (17,867). Consequently, the patient received meropenem hydrate 3 g/2 days as empiric antibiotic treatment for acute infection (Fig. 2).

**Figure 3.**
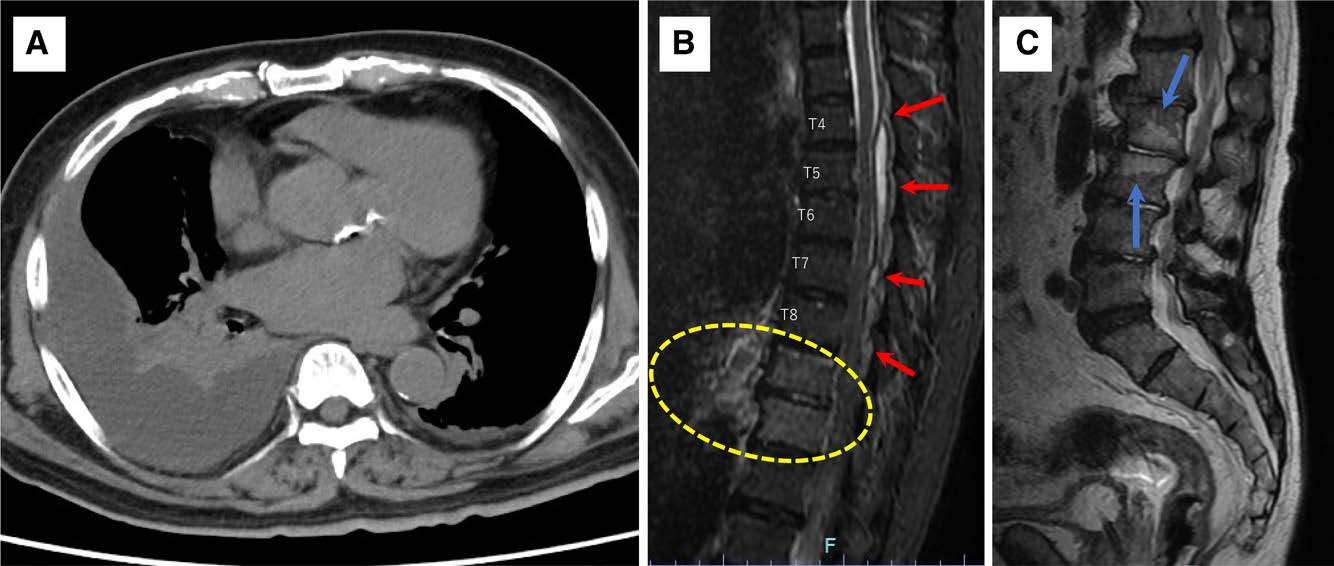
Image of the abscess and spondylodiscitis at intensive care unit transfer. (A) Chest computed tomography image showing dense right pleural effusion. (B) MRI (STIR sequence) showing abscess formation in the T4–8 regions (red arrowhead); yellow dotted circles denote spondylodiscitis and edematous changes in the surrounding soft tissues at the T9–10 regions. (C) MRI (T2-weighted sequence) showing spondylodiscitis between the L2–3 regions (blue arrowheads). MRI = magnetic resonance imaging, STIR = short tau inversion recovery.

Upon admission to the emergency department, the patient Glasgow Coma Scale score was as follows: eye opening, 4; best verbal response, 5; and best motor response, 6. Lower extremity manual muscle testing yielded scores of 0 to 1 and bilateral Babinski reflexes were present. Sensory perception was reduced in T10 dermatomes and lower limbs. Contrast-enhanced magnetic resonance imaging and computed tomography (CT) were performed to identify the infection source, revealing the presence of right empyema and pyogenic spondylitis involving the Th4–8 regions (Fig. 3A and B). Spondylodiscitis was observed in the T9–10 and L2–3 regions, with contrast enhancement suggestive of inflammatory edema, particularly in the ventral soft tissue of the T9–10 region (Fig. 3B and C). The patient was scheduled for surgical removal of the infected tissue and was eventually transferred to the intensive care unit (ICU) on POD 16. Empyema drainage was performed on the same day, followed by epidural empyema drainage and left hemisectomy in the T4–8 regions on 17 POD. Blood cultures, sputum tests (upon emergency department admission), and cultures from the thoracic and spinal abscesses drained during surgery revealed MSSA. Following thoracic and spinal drainage, the inflammatory marker levels and respiratory symptoms steadily improved. The patient was transferred from the ICU to the general ward on 19 POD. Preoperatively, the patient maintained good oral function (including mastication and swallowing) and received professional oral care. Based on previous studies recommending at least 8 weeks of antimicrobial therapy for spondylitis,^[12,13]^ and the delayed normalization of CRP levels, the antibiotic regimen was switched to cefazolin sodium hydrate at 6 g/2 days on 22 POD, which was continued until 72 POD.

**Figure 4.**
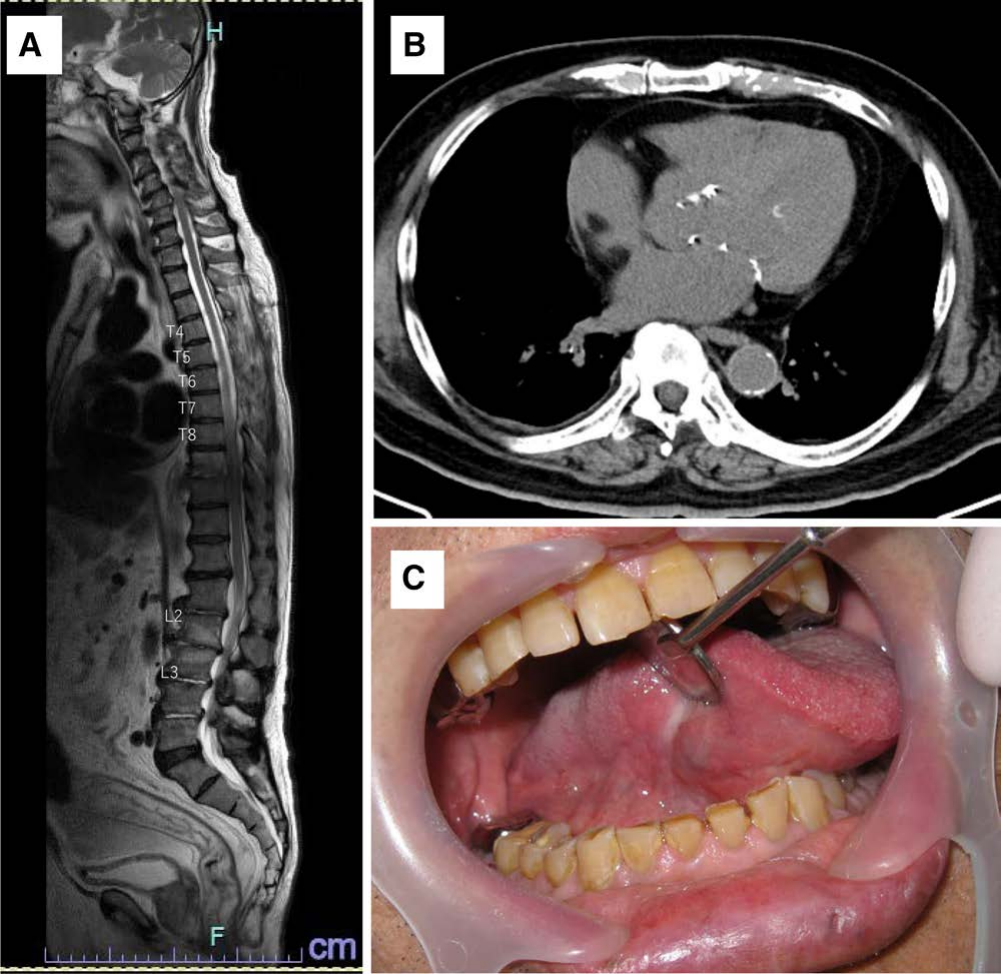
Imaging findings at the completion of treatment. (A) MRI (T2-weighted sequence) shows improvement in inflammatory findings throughout the spine, with no apparent abscesses. (B) No abscesses are observed. (C) No tongue cancer recurrence is observed. MRI = magnetic resonance imaging.

Drainage and long-term antimicrobial therapy successfully eradicated the pathogens and resolved inflammation. The patient was transferred to a rehabilitation hospital on 45 POD to facilitate the recovery of lower extremity motor function. Figure 4 shows the current pulmonary, spinal, and oral assessment findings, which indicate the absence of abscesses or recurrence. The patient continued to undergo functional restoration training, gradually regained function, and eventually achieved the ability to walk with grasping gait.

## 3. Discussion

Our literature review identified no previous case of SA-induced pyogenic spondylitis with empyema. A PubMed search was conducted using the following Medical Subject Headings terms: “spondylitis” OR “spondylodiscitis,” AND “empyema and pleural.” We reviewed the data of 11 patients to determine the causative organisms of both the diseases. *Fusobacterium spp*. and *S milleri* were identified in some patients.^[4,14,15]^ *Brucella* was the causative agent in 1 patient with concomitant spondylodiscitis and empyema.^[16]^ Notably, no previous studies documented SA as the culprit. Although a few cases of secondary spondylodiscitis following oropharyngeal surgery and poor oral hygiene have been reported,^[1,17]^ no study has reported the concomitant occurrence of spondylitis and empyema after oral surgery.

The global COVID-19 pandemic, including a surge in critically ill patients admitted to our hospital in Japan,^[18]^ has necessitated shorter hospital stay. Typically, discharge occurs when the surgical wound heals and the patient can independently perform oral hygiene. However, in this case, the patient was discharged on 3 POD even if the wound had not completely healed, potentially increasing the risk of infection owing to a lack of oral hygiene management. Therefore, we strongly suspected that MSSA, an endemic oral bacterium, was hematogenously transmitted to the spine from either the extraction fossa or wound during tongue cancer resection. Endotracheal intubation during surgery is a potential route of infection for patients with lung abscesses. However, considering that the disease onset coincided with the time of pyogenic spondylitis occurrence and that the same bacterial species were detected, it may be more reasonable to assume that the lung abscess was attributed to the same hematogenous infection. In this case, several factors increased the risk of hematogenous infection. Oral steroids for the treatment of polymyalgia rheumatica and DM can suppress immunity and increase the susceptibility to infections. Early hospital discharge before regaining adequate oral hygiene skills may have exacerbated the already compromised oral hygiene, allowing MSSA to invade unhealed wounds and enter the bloodstream. Supporting this notion, Yoshizawa *et al* demonstrated that poor oral hygiene was correlated with increased detection of pneumonia-causing bacteria in the blood, even in patients with concomitant sepsis and meningitis caused by *Streptococcus constellatus*, an endemic oral bacterium, following a dental procedure.^[5,19]^ Furthermore, odontogenic infections are a significant risk factor for pyogenic spondylitis, with MSSA, the same causative species as in this case, being the most commonly detected.^[2,20]^ Preoperative oral cleaning reduces the risk of postoperative complications in various types of cancer.^[21]^

This case emphasizes the importance of meticulous oral care and maintenance of good oral hygiene to prevent postoperative complications and maintain high quality of life. Early diagnosis and prompt treatment are crucial in suspected hematogenous infections. This involved comprehensive medical history, targeted blood tests, and whole-body contrast-enhanced CT scans. Early diagnosis of pyogenic spondylodiscitis or empyema, followed by the administration of appropriate intravenous antibiotics and drainage procedures, is vital to avoid severe neurological sequelae such as spondylodiscitis and lethal respiratory failure.

## 4. Conclusion

This case underscores 2 essential lessons for readers. First, following surgery, patients should be thoroughly educated to seek medical attention upon experiencing subjective symptoms, such as torso pain or difficulty walking. Early evaluation is crucial to rule out potential complications, such as spondylitis or empyema. Second, the collaborative efforts of oral surgeons, ward nurses, neurosurgeons, respiratory surgeons, and ICU staff who performed necessary examinations and procedures reduced the risk of functional impairment after transfer to the ICU. This case highlights the critical role of close collaboration among medical teams, particularly in the management of rapidly progressing systemic diseases.

## Acknowledgments

We would like to thank Editage for a meticulous review of the English language used in this manuscript.

## Author contributions

**Conceptualization:** Kunio Yoshizawa, Akinori Moroi.

**Investigation:** Kunio Yoshizawa, Takashi Yagi, Tsuyoshi Uchida, Takeshi Moriguchi.

**Supervision:** Takashi Yagi, Tsuyoshi Uchida, Takeshi Moriguchi, Koichiro Ueki.

**Writing – original draft:** Kunio Yoshizawa.

**Writing – review & editing:** Kunio Yoshizawa, Takashi Yagi, Tsuyoshi Uchida, Takeshi Moriguchi, Akinori Moroi, Koichiro Ueki.
